# 18F-FDG PET/CT Imaging of Gallbladder Adenocarcinoma - A Pictorial Review

**DOI:** 10.7759/cureus.298

**Published:** 2015-08-09

**Authors:** Faiq Shaikh, Omer Awan, Salman A Khan

**Affiliations:** 1 Imaging Informatics, University of Pittsburgh Medical Center; 2 Molecular Imaging Physician, S&L Readings, LLC.; 3 CEO, Crunchtimr Medical Solutions, LLC; 4 Department of Radiology, Dartmouth Hitchcock Medical Center; 5 Department of Internal Medicine, University of Missouri Kansas City

**Keywords:** PET/CT, fdg pet, mri, gallbladder adenocarcinoma

## Abstract

Gallbladder adenocarcinoma is an uncommon and serious disease. The primary disease grows rapidly with local invasion into the liver and with distant spread to lymph nodes. It is often detected late, due to which management can be challenging. Despite routine use of computed tomography (CT) and ultrasonography (US) for detection, magnetic resonance imaging (MRI) is often considered for a detailed assessment of the anatomic behavior of these tumors. We share three cases where 18-FDG PET/CT played a role in management thereof.

## Introduction

We share our experience with management of this rare disease, from a multimodality imaging standpoint. At our medical center, a total of 67 patients were identified between January 2003 to June 2011 with the diagnosis of gallbladder cancer. Ten of these patients underwent a PET/CT scan during the course of their management, three of whom had positive PET/CT findings. Two of these cases had positive MRI correlation.

## Case presentation

Informed patient consent was obtained from all participants described in this report prior to treatment.

### Case 1

**Figure 1:**
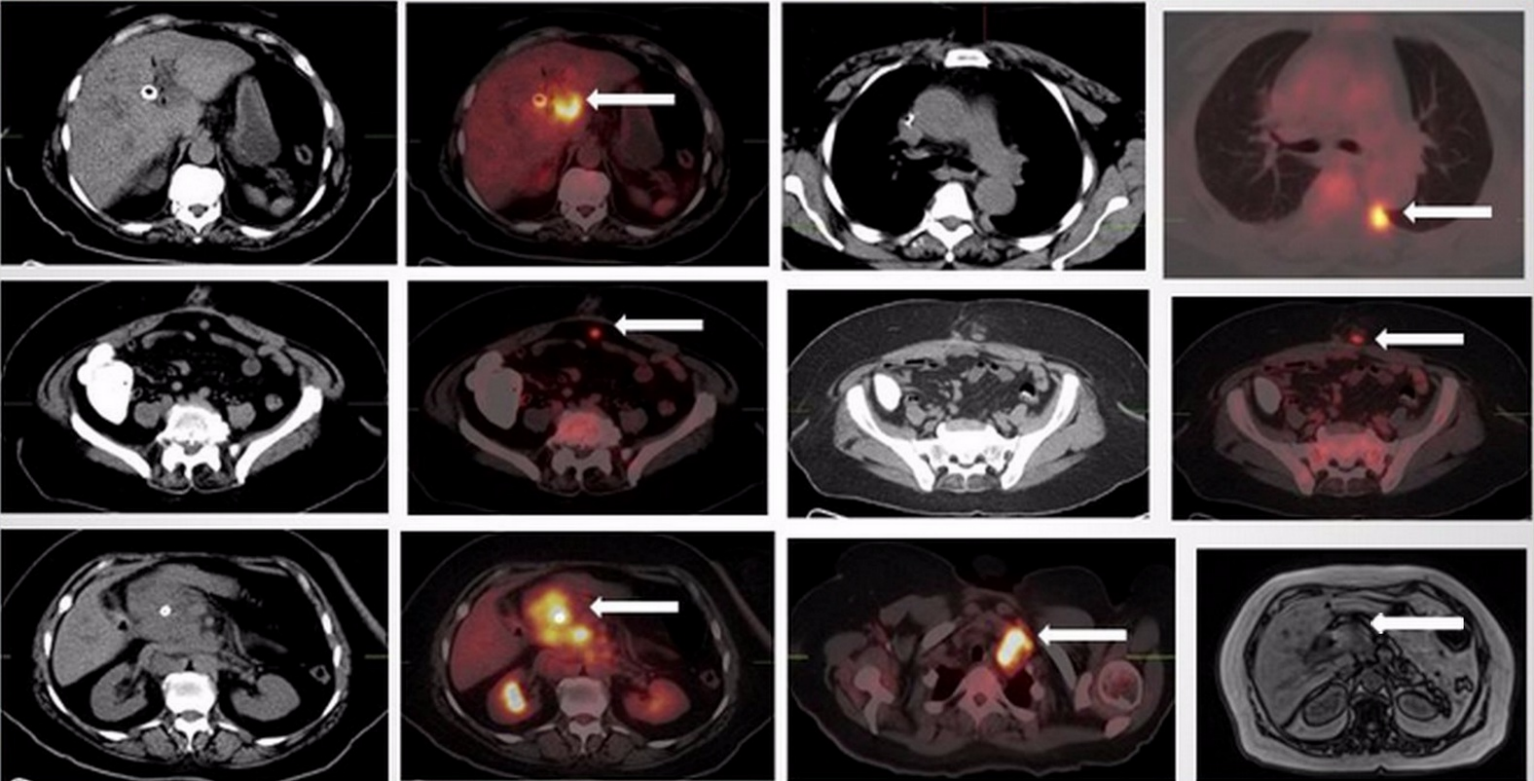
FDG-PET/CT and abdominal MRI PET/CT images demonstrate an irregular hypodense left hepatic lobe lesion, right posterior pleural-based density, deposits in the midline anterior abdominal cavity adjacent to the umbilical port site, soft tissue density in the subcutaneous tissue of the anterior abdominal wall superficial to the umbilicus, a poorly-circumscribed infiltrative epigastric mass with a centrally located biliary stent and multiple lymph nodes in the left supraclavicular station with increased FDG uptake (see arrows). The abdominal coronal T1-weighted VIBE MR images demonstrate a low-intensity left hepatic lobe lesion that is also seen as an infiltrative mass on axial MR images (see arrow).​

A 60-year-old female presented with two years of vague nonspecific symptoms of nausea, vomiting, and left flank pain. A right upper quadrant abdominal ultrasound was performed that demonstrated calculous cholecystitis with mild intrahepatic ductal dilatation, for which she underwent cholecystectomy. The surgical pathology confirmed gallbladder adenocarcinoma. A follow-up MRI was performed at an outside institution that reported post-surgical cholecystectomy changes and no evidence of metastatic disease. A month later, the patient developed a common duct stricture requiring placement of a biliary stent. Subsequently, she received an MRI that demonstrated extensive abdominal lymphadenopathy, but the surgical bed and the pancreatic head were obscured by the metallic artifact from the biliary stent placed, as seen on coronal T2-weighted HASTE and axial T1-weighted VIBE images. Alternatively, a PET/CT was performed that demonstrated an FDG-avid right hepatic hypodense lesion where the biliary stent was noted. Additionally, a left lung pleural implant with increased FDG uptake was noted, along with FDG-avid periumbilical intra-abdominal and periumbilical midline abdominal wall implants that were suggestive of possible surgical tract seeding. Also noted was an FDG-avid right supraclavicular node (Figure 1). Based on these findings, the cancer was deemed Stage IV and the patient a poor surgical candidate. However, chemotherapy was deferred due to poor functional status and the patient was referred for hospice care.

### Case 2

**Figure 2:**
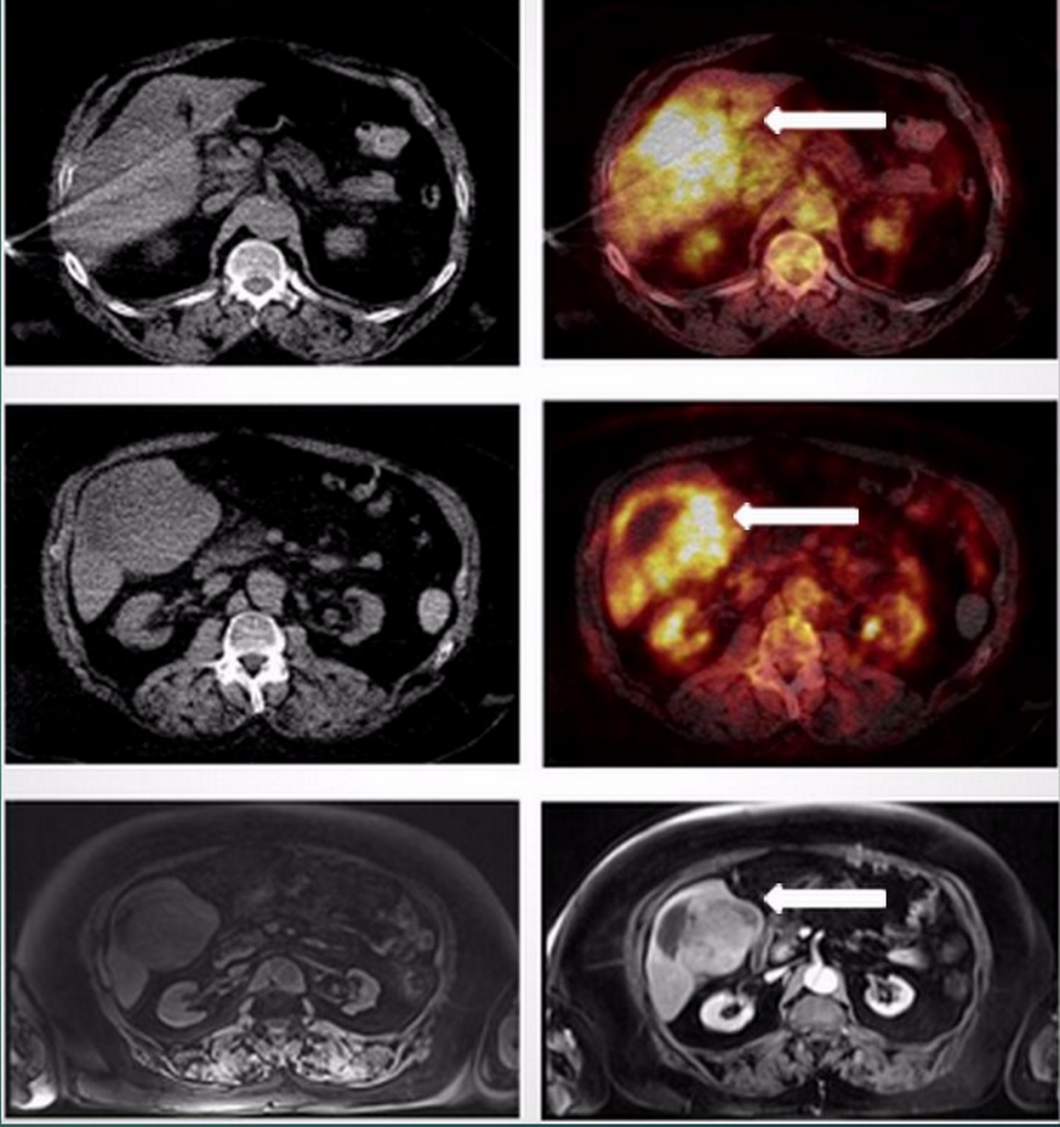
FDG-PET/CT and abdominal MRI The PET-CT study demonstrates an infiltrative right inferior hepatic lobe lesion with increased FDG activity (see arrow). There is increased T1 signal with heterogeneous increased T2 signal and heterogeneous post-contrast enhancement on axial T1-weighted VIBE images (see arrow).​

A 65-year-old male presented to emergency room with features of biliary colic and was found to have a large heterogeneous mass on a right upper quadrant ultrasound. Further evaluation with an abdominal CT demonstrated an enhancing heterogeneous mass associated with mild intrahepatic ductal dilatation. MRI confirmed a T1 10.6 x 11.0 x 9.1 cm mass gallbladder fossa mass demonstrating decreased T1 signal with heterogeneous increased T2 signal and heterogeneous post-contrast enhancement on axial T1-weighted VIBE, coronal T1 weighted, and coronal T2-weighted HASTE and post-contrast T1-weighted VIBE images. The mass was seen to be infiltrating the medial left hepatic lobe and anterior division of the right hepatic lobe. A PET/CT was performed to assess for distant metastatic disease and demonstrated a heterogeneously dense right hepatic lobe lesion with increased FDG uptake on fusion images. Also seen was a hypodense region in the inferior right hepatic lobe with decreased FDG uptake on fusion images, suggestive of necrosis, but no metastatic disease was detected (Figure 2). Despite the latter, based on the infiltrative pattern of the primary lesion on both PET and MRI, the primary cancer was considered to be borderline unresectable. Additionally, given the associated comorbidities, surgery was deferred, and chemotherapy using Gemcitabine was initiated instead.

### Case 3

**Figure 3:**
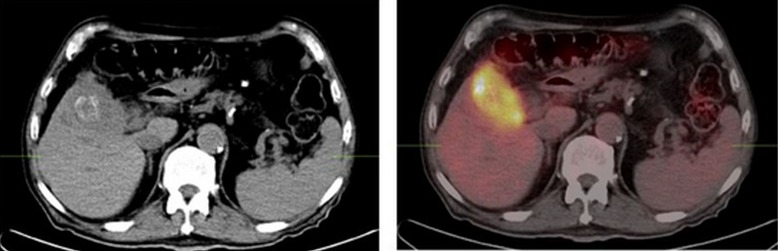
FDG-PET/CT These images demonstrate increased FDG uptake in the ​primary lesion infiltrating the liver.​

An 85-year-old male was referred to the outpatient clinic with jaundice and weight loss. A CT of the abdomen showed biliary dilatation, and a partially calcified, mildly hypodense gallbladder mass infiltrating the liver. A PET/CT study demonstrated increased FDG uptake in the primary lesion infiltrating the liver that was felt to be marginally resectable, but with no evidence of metastatic disease (Figure 3). Again, given the imaging-based evidence of local infiltration of the primary tumor, surgery was deferred despite the absence of metastatic disease, and chemotherapy with Gemcitabine was initiated.

## Discussion

The incidence of gallbladder cancer in the USA is approximately 9,700 cases per year [1]. More than 80% of these cases are detected late while in advanced stages with either locally aggressive or advanced metastatic disease (Stage III and up), primarily due to vague presenting symptoms such as fever, jaundice, and abdominal pain. Approximately 80% of the time, it is discovered incidentally on post-cholecystectomy pathologic analysis. The five-year survival rate for Stage I disease is 50%, and less than 10% for Stage III and above (with metastatic disease). Chronic inflammatory disease, obesity, environmental toxins, heavy metal, and radiation exposure are predisposing factors for this malignancy [1].

Currently, the management options for gallbladder cancer include surgery (cholecystectomy with partial hepatectomy), chemotherapy, radiation therapy, and palliative endoscopic stenting in inoperable cases [2]. Ultrasonography, CT, and MRI/MRCP are routinely performed for the purposes of detection, staging, and restaging of this disease.

Fewer than 2% of patients with gallbladder cancer currently receive FDG-PET/CT during the clinical course [3]. For the purpose of staging, PET/CT has similar sensitivity (90%) to CT for primary disease (unless T1); however, the sensitivity is much more superior (90%) as compared to that of CT (67%) for T1 and metastatic disease. For restaging after cholecystectomy, PET/CT provides the added benefit of detecting metastatic seeding of the portal tract with high sensitivity, which can be very difficult to detect on other modalities [3-6]. While abdominal MR imaging can be effective in evaluation of local infiltration of the primary gallbladder cancer into the surrounding liver parenchyma (100%) as well as lymph node invasion (92%) [7], it may be limited by metallic artifact from the cholecystectomy surgical clips or a biliary stent [8], which are frequent in this patient population. In such cases, PET/CT may alternatively provide better assessment due to a milder metallic artifact on the CT portion and with the help of uncorrected images for assessment of FDG uptake in those regions. In addition, quantitative assessment using standardized uptake values (SUV) can be useful in the follow-up PET/CT after chemotherapy to assess response, although this needs to be validated further.

Our cases demonstrate the usefulness of PET/CT in the assessment of the direct invasion by the primary gallbladder cancer lesion, as well as evaluation of distant metastatic disease. Most of the cases were incidental gallbladder cancers diagnosed postoperatively on surgical pathology. PET/CTs performed in these patients not only confirmed the findings seen on staging CTs but also detected new distant metastatic lesions. They were useful in guiding the decision of initiating chemotherapy in patients with locally infiltrated or advanced metastatic disease. PET/CT can be a useful modality for assessment of disease response to chemotherapy when compared to pre-therapy PET/CTs. We had one case where the MR images were suboptimal due to the artifact from a biliary stent, but the extent of local infiltration, as well as distant disease, was adequately evaluated using PET/CT.

## Conclusions

Little has been reported on the use of FDG-PET/CT imaging in gallbladder adenocarcinoma, and based on our limited experience, we believe that PET/CT can be a useful modality for staging and restaging of this malignant disease, therapy response assessment, and may be a viable alternative to MRI in patients in the assessment of local invasion with post-procedural metallic stent placement.
